# Cincinnati Academy of Medicine

**Published:** 1881-04

**Authors:** 


### CINCINNATI ACADEMY OF MEDICINE.

---

*Dislocation of the Hip-Joint.*—Dr. Reuben A. Vance read the following essay on dislocation of the hip:

The questions involved in dislocations of the hip are neither few nor unimportant. I doubt if there is any topic in surgery of equal gravity in which there is such diversity of opinion or contradiction in teaching. It is needless to inquire the cause. The complexity of the parts in which the lesion occurs, the different relations assumed by the bones of the thigh and pelvis in sitting, standing and walking, together with the widely unlike circumstances under which dislocations of the hip occur, the readiness with which the ordinary anatomical landmarks can be obliterated by improper movements of the injured extremity and the infrequency with which post-mortem examinations are made in individuals who have suffered this injury, all go to show why this state of facts should exist. Unfortunately this branch of surgery—in fact, this particular lesion—has been a favorite field for a class of theorists who adduce opinions without facts upon which to base them and advance inferences which more frequently come from their imaginations than their observations. In order to understand dislocations of the hip the anatomy of the part must be studied by actual dissection, recorded cases analyzed, the lesion itself investigated and morbid specimens examined. In a word, it is from clinical and pathological investigations that the rules for the interpretation of recorded cases are to be derived, and by converging the rays of fact emerging from each of these sources, surgeons will ultimately be enabled to illuminate this topic with the light of truth.

I desire this evening to contribute my mite to the solution of those problems which must be unraveled before dislocations of the hip can be robbed of the terrors they possess for many practitioners. In view of what I have just said as to certain assumptions that have been advanced elative to the pathology and treatment of this lesion, it may not be improper for me to allude to the facts upon which the inferences I shall draw are based.

Seventeen years ago, while a student, I was by the fortunes of war forced to remain several hours with a fellow-soldier who had dislocated his hip. The position of the limb, the aspect of the patient, and the measures resorted to for the cure of the luxation made an indelible impression on my mind. A veterinary surgeon from a passing cavalry regiment was the first individual with pretensions to professional knowledge to come and tender assistance, and by his manipulations the dislocation was reduced. In subsequent conversations with a regimental surgeon of no mean attainments, this subject acquired an interest that has since led me to investigate the matter at all times and under every circumstance where it was possible to make inquiry as to the pathological character of dislocations of the hip, or to study the procedures adopted for their reduction. Aside from personal experience with patients thus injured, I have had an opportunity to study the preparations illustrative of this lesion in two hospitals in New York City, in the Pathological Museums of Dublin, Edinburgh and London, and in several private cabinets.

The first patient in whom I reduced a dislocated hip died eight weeks subsequently of an intercurrent affection, and I was permitted to make a thorough examination of the part, although not allowed to remove the specimen. In another case, in which dislocation occurred, and was immediately reduced, in 1862, the patient dying in 1876, I made a similar inspection. These circumstances, in connection with the fact that I have either given written opinions or testified orally in seven suits at law, arising from what in each instance was claimed to be a dislocation of the head of the femur, will show that I have not been inattentive to the literature of the subject in either its surgical or legal aspects. In 1865–66 it was my good fortune to listen to the surgical lectures of Moses Gunn, then professor of surgery in the University of Michigan, now occupying a chair in Rush Medical College, Chicago, and his clear demonstrations solved the doubt that for more than two years had enveloped this subject. To my friend, the regimental surgeon before alluded to, the case of the soldier was clear enough so long as I confined myself to an account of the flexed, adducted, inverted and shortened limb, but so soon as I said that the veterinary surgeon, in his successful attempt at reduction, first bent the leg on the thigh, the thigh on the body at the same time pulling tke knee and carrying it more and more across the other thigh, until, with an audible snap, the head of the bone returned to its socket, the Doctor was all at sea. So soon as I made that statement, and insisted on its accuracy, he would declare, with no little emphasis, that there was a mistake somewhere, and that no such manipulation could reduce a dislocation of the character described. It was in vain that I studied the anatomy of the part and sought an explanation in surgery after surgery. I found no solution of the anomaly until I listened to Gunn's lectures. The peculiar circumstances narrated gave me an interest in this lesion that led me, while House Surgeon of Bellevue Hospital, New York City, to do missionary work in the dissemination of Gunn's ideas. In this I was materially aided by a case in which I not only reduced the dislocation of the hip in the presence of the resident staff, but had an opportunity of confirming the diagnosis and investigating the state of the parts by a post-mortem examination.

I embodied what I considered Gunn's ideas, in a paper, and read it before the Bellevue Hospital Medical Union. In this paper were the details of three cases, in which the method employed was based on the principles taught by Gunn; in each case the dislocation had been reduced readily and painlessly; and in one instance the result of the post-mortem was appended, the patient having died of pneumonia.

Between 1867 and 1872, I was busily engaged in public and private medical teaching, and as material at my disposal was ample, I was enabled to demonstrate upon the cadaver the mechanism of this lesion in its various forms, and to illustrate the manipulations requisite for the reduction of each variety. The explanations given were so different from those usually taught, the demonstratians so complete and satisfactory, and the treatment so effectual and withal so reasonable, that I had no difficulty in arousing a degree of interest in the subject in the minds of my students that, in time, yielded me a rich harvest in the line of personal experience in dislocations of the hip. I make these personal references in order to exhibit the source of the facts—anatomical, clinical and pathological—

from which are deduced the conclusions I now desire to propound for your consideration.

The acetabulum is not equally strong at all points, but its osseous wall and cartilaginous and fibrous rim are so adapted to each other that at those points where the greatest impacts are received and the most powerful pressures exercised you find the excavation deepest and the osseous and cartilaginous embankment the highest. The cotyloid notch is the weakest part of the acetabulum. This is directly downwards and slightly backwards when the pelvis is in the position assumed during the erect posture, and is closed by the transeverse ligament. As we pass from the osseous and cartilaginous to the ligamentous structures of the hip, we find precisely the same law in operation; where the greatest tension is habitually exercised, there the ligamentous structures are best developed. The capsular ligament illustrates this principle admirably. In this structure the fibres pass from the brim of the acetabulum to the neck of the femur, a few strands coming from the anterior inferior spinous process of the ilium, and a few from the anterior aspect of the ilio pectineal eminence. An unbroken continuity of fibres can be traced from the pubic border of the cotyloid notch upwards, backwards and downwards to the ischiatic border of the cotyloid notch. These fibres are thickest on the anterior aspect of the neck of the femur; those above and behind come next, while the weakest portion of the capsule is the part connecting the inferior aspect of the femoral neck with the transverse ligament. Before any conclusions are drawn from these facts another circumstance should be taken into consideration: are there any structures in the neighborhood that tend to prevent the direction of the force against the weak point? Although the part of the capsule continuous with the transverse ligament closing the cotyloid notch is the weakest portion of that structure, yet the situation and action of the powerful adductor muscles so protect it that luxation at this point is one of the rarer forms of dislocation of the hip. Weak as is this portion of the capsule, a luxation here can only occur during abduction, and the powerful adductor muscles, preventing that movement, keep the head of the femur from impinging on the under portion of the capsule. When abduction does occur, the head may penetrate its coverings just without the transverse ligament—a condition that will be dwelt upon in another part of this paper. The anatomy of the joint and surrounding parts demonstrates this fact: if the head of the femur be brought in contact with the capsule at any point, and sufficient force applied, a dislocation may result.

An examination of specimens, and a careful study of clinical phenomena, convinces me that not only may you have a dislocation at any point as anatomy teaches, but that in point of fact you do not have the head of the femur forcing its way through the capsule at the point it chances to occupy when sufficient force is applied. The force that sends the head of the femur through the capsule, however, in the vast majority of cases, simply carries the articular extremity out of its socket, and does not tear away that part of the capsule opposite the opening. If the circle of the brim of the acetabulum be divided into 360 degrees, it could be truthfully said that dislocations occur at every degree. The symptomatic phenomena characteristic of dislocations—the flexion, inversion or eversion, shortening or lengthening, abduction or adduction, etc.—are mainly due to the portion of the capsular ligament which has not been ruptured by the force that dislocated the limb. The capsular ligament gives way at the point where the head impinges upon it at the critical moment; in the majority of cases there is a simple slit in the capsule, or at most, a straight button-hole slit, conjoined with a rupture of a few fibres at the acetabulum, which give to the capsule opening a "T" shape. As this opening will but rarely occur in exactly the same place in any two cases, the symptoms developed by the traction exercised on the limb by the untorn portion of the capsule will seldom be alike in any two cases. Consequently what are designated "classical" or "regular" dislocations are but typical illustrations of the more commonly recurring forms of this lesion.

In order to comprehend the measures necessary for reducing a dislocation, it must be borne in mind that certain movements of the lower extremity are associated.

Thus, when abduction occurs, this movement is naturally accompanied by eversion; adduction by inversion, etc. A man falling from an elevated point to the earth flexes his legs upon his thighs, his thighs upon his body, and adducts his knees; that posture is instinctively assumed in which the greatest amount of force can be received by the feet with the least damage to the organism. Should the force be greater than can be disposed of without harm, a dislocation may be produced. If the thigh be extended and strongly adducted, the head of the femur passes directly upwards through the top of the capsule. It may now assume one of three positions: it may remain with the head hanging over the acetabulum; the head may advance and the great trochanter recede; or the head may recede and the great trochanter advance. The dislocating force perforates the capsule and carries the head upon the brim of the acetabulum; the neck is surrounded by the borders of the rent in the capsule and held firmly by the untorn portion of that structure—the subsequent movements are accidental and may or may not occur. If the thigh be flexed and adducted at the moment force is applied, the position of the head of the bone will vary with the amount of flexion and inward rotation. As before, so here, the neck will be held by the untorn portion of the capsular ligament, and the deformity that results will be mainly due to the tension it exercises. Should the force be applied while the limb is abducted dislocation downward occurs. Should the thigh be hyperextended, adducted and rotated outwards, force, directly or indirectly applied, will carry the head of the femur forward upon the pubes. Should it be forcibly flexed upon the body, adducted and rotated inward, it will emerge directly opposite the tuberosity of the ischium and may rest on that prominence. Through injudicious manipulation of the dislocated extremity the head of the femur, when dislocated directly downwards, may be carried towards the pubes or the ischium, and in certain cases where force is used it may even recede behind the ischium and pass without the internal obturator tendon. Between these typical and well defined dislocations there are innumerable intermediate forms insensibly shading off into each other.

If the surgeon desires to demonstrate the influence of the capsular ligament upon different dislocations, he should take a subject and on one side remove all the tissues about the hip-joint except the capsular ligament, and on the other make a subcutaneous section of the capsule, cutting as little as possible of adjacent structures. In the former case, by making longitudinal slits in the capsular ligament, he can produce any form of dislocation he pleases and study the essential symptoms—lengthening or shortening, adduction or abduction, flexion, inversion, etc.—thus developed at his leisure. In the latter case the limb can be readily put in or out of joint, but the symptoms marking the different forms of luxation of the head of the femur are wanting.

The principles which should govern the surgeon in his efforts at reduction can be thus formulated:

1. Place the limb in the position it occupied at the moment it forced its way through the capsule, thus carrying the head of the femur opposite the opening through which it emerged.

2. Manipulate the limb in such a manner as to relax the untorn portion of the capsular ligament.

3. Draw or push, elevate or depress the head of the femur in such a manner as to carry it over the brim of the acetabulum, excrcising this force by proper movements of the extremity, directed by the grasp the surgeon has on the leg, at the same time so moving the limb as to keep constantly relaxed the untorn portion of the capsular ligament.

How are these principles to be applied? For example, how can the surgeon determine the position the limb occupied at the moment the head of the femur forced its way through the capsular ligament? A knowledge of anatomy and an acquaintance with the mechanism of hip-joint luxations, together with the history of the given case and careful study of the signs and symptoms presented by it, will almost invariably supply all needed information. The axis of the femur will direct the surgeon to the immediate region of the opening in the capsular ligament; the position of the head of the bone or the situation of the great trochanter will reveal the extent and character of any movement of the articular extremity that occurs subsequently. In dislocations upwards, or upwards and backwards or backwards and downwards, but little difficulty will be had in determining this point in the case. The same is true of downward dislocations. Dislocation on the pubes or on the tuberosity of the ischium, owing to the part borne by inward and outward rotation in their mechanism, must be carefully studied before any attempt is made to reduce them, in order to distinguish between these lesions and the changed position of the head of the femur in certain downward luxations. The application of these principles in detail can be best illustrated by describing their application to a few typical cases. In describing symptoms the patient is presumed to stand erect; in detailing manipulations for reduction, to be recumbent.

In the case of a patient whose left thigh was dislocated by a blow from a runaway horse, the femur was forcibly adducted while extended. The head of the bone could be felt behind the spinous processes of the ilium and upon rotating the foot outwards—consequently the thigh inwards—at the same time flexing the leg on the thigh and adducting the knee, the round articular extremity receded and the great trochanter advanced. When first seen the left lower extremity was shortened, adducted and flexed; the left thigh crossing the right just above the knee. Upon adducting the thigh and rotating it slightly inwards, the head of the femur receded and the trochanter advanced. When this movement was continued until firm resistance was experienced, the head disappeared and the great trochanter occupied a position just behind the place first assumed by the head. In a word, the neck of the femur was in the grasp of the ruptured capsular ligament and the head and great trochanter were the opposite extremities of a lever, the centre of revolution of which was at the opening in the capsule through which the head had emerged. The following maneuvres were adopted for the reduction of the luxation: Standing on the right side of the patient, I grasped his left ankle with my right hand and his left leg just below the knee on its posterior surface with my left hand; then flexing the leg upon the thigh I adducted the latter slightly, at the same time rotating the femur (by acting on the left leg with my right hand) until the great trochanter was directed outwards and the head of the bone inwards; next, the movement of adduction of the thigh was resumed, at the same time making traction in the line of the axis of the femur with my left hand, while short but rapid from side to side movements were communicated to the left ankle with my right hand. There was a progressive advance of the head of the femur from the point it occupied after being directed inwards until it reached the margin of the acetabulum; here it halted for a few seconds and then with a sudden forcible impulse it snapped into its socket.

When the thigh is flexed as well as adducted at the moment the luxation happens the head of the femur may occupy any point between the situation just described and the ischiatic notch. Generally the head of the bone is not far from the opening in the capsule through which it emerged—usually is a little above it. In order to return the more or less shortened, adducted, flexed and inverted limb to its socket, the following manipulations are necessary: Let the surgeon stand on the uninjured side and grasp the ankle and leg as before, flexing the leg on the thigh and rotating the femur a trifle inwards, and execute simultaneously the triple movement of adduction, firm traction in the axis of the femur, and flexion of the thigh, at the same time communicating to the ankle the vibratory movements above described. In order to appreciate the movements thus given the dislocated part, we must picture to ourselves the situation of the head of the femur and acetabulum. At the moment of dislocation the leg was flexed on the thigh, the thigh on the body and the knee adducted. But the head of the femur does not remain opposite the point at which it emerged; almost invariably the femur is rotated outwards, abducted and extended as far as the untorn portion of the capsule will permit. The flexion of the leg on the thigh relaxes the hamstring muscles, the inward rotation of the fermur carries the long axis of the head and neck of that bone in the direction of the long axis of the opening in the capsule, while the combined movements of adduction, traction upon and flexion of the thigh relaxes the tense untorn portion of the capsule and carries the head of the bone upon the edge of the acetabulum, and against the slit in the capsular ligament, but apparently finds further progress impossible from the small size of the opening in the capsule; if now the surgeon communicates to the ankle slight to and fro movements, almost instantly such an amount of reflex contraction is excited, in the adductor muscles of the thigh, as suffices to forcibly draw the head of the femur into the acetabulum.

Should the dislocation be downward and forward upon the thyroid foramen, a very striking deformity ensues: the limb is lengthened, abducted, advanced, inverted and flexed. To effect reduction in this case, the leg is semi-flexed on the thigh, the thigh abducted, slightly flexed on the abdomen and strongly drawn upon in the line of its long axis. Should the head of the femur ascend to the rim of the acetabulum and there meet with resistance, it should be held in this situation for a moment while slight to and fro movements are communicated to the ankle—this maneuver excites sufficient reflex muscular contraction to draw the bone into its socket In reducing this lesion the surgeon stands on the injured side and grasps the leg as before.

The foregoing are the ordinary varieties of luxation of the hip. If inward or outward rotation be combined with forced adduction, the articular extremity of the femur will be carried forward on the pubes or backwards on the ischium. The same principles govern their reduction that apply to the foregoing varieties—in any case of luxtion at the hip the surgeon should endeavor by relaxing the untorn portion of the capsular ligament and carrying the head of the bone to the orifice through which it emerged to effect a reduction.

Finally, in dislocations upon the thyroid foramen or tuberosity of the ischium, careless or ignorant manipulation may carry the head of the bone beyond the tuberosity and without the tendon of the internal obturator muscle. Here is a dislocation closely resembling one form of the upward and backward variety that by certain writers is claimed to be by no means infrequent. In one case, under my observation, the leg was flexed on the thigh, the thigh adducted, flexed and drawn across the middle of the right thigh and so rotated inward that the sole of the foot pointed outwards and forwards. The accident occurred in jumping off the street cars—the left leg was flexed and forcibly adducted. A man came to his assistance, who, finding the thigh directed forwards and the leg flexed on the thigh and projecting from it laterally, caught the knee and ankle and gave the leg a twist which resulted in the above described condition. Reduction was effected in this way: Standing on the uninjured side I grasped the left leg in the usual manner, the thigh was forcibly but slowly adducted at the same time the femur was rotated outward, drawn upon in the line of its long axis and flexed on the abdomen. These movements carried the head of the femur to the brim of the acetabulum, the usual to and fro motion was given the ankle, and at once the bone returned to its socket.

The opening in the capsular ligament being of the slit-like nature already described, anyone taking the trouble to study this question upon proper preparations can convince himself, not only as to what it is that holds the limb in the peculiar position it occupies in each of the luxations and of the efficacy of the various steps described in reducing these dislocations upon the principles just portrayed, but he can also satisfy himself of the unphilosophical character of the special manipulations described by Reid. In dislocations upon the dorsum ilii Reid's method consists in "flexing the leg upon the thigh, carrying the thigh over the sound one upwards over the pelvis as high as the umbilicus, and then abducting and rotating it." Notice the fatal defect: "If the dislocation is directly upwards any "carrying the thigh over the sound one upwards over the pelvis as high as the umbilicus" will necessarily fail to reduce the dislocation, from the fact that this manipulation does not relax the untorn portion of the capsular ligament, and does not carry the head of the bone to the opening through which it emerged. Should the dislocation be upwards and backwards, these manipulations are, if anything, even more palpably inefficient, and for the same reasons. But to the two final movements communicated to the injured extremity, a more forcible objection can be made—they are not only inefficient, but they may be very injurious. Picture to yourself the situation of the parts: The preceding manipulations move the head of the bone in its abnormal situation, and do not carry it to the opening in the capsule and so relax the tense structures as to permit reduction; these final movements of abduction and rotation simply exert powerful traction on the untorn portion of the capsule, and unless (as very frequently happens) a compensatory movement of the spine occurs, thus protecting the capsule, certain of the fibres of the latter must be lacerated. Many surgeons have reduced dislocations of the hip who thought they were using Reid's method, yet a careful study of the principles involved, will show that their success was due to other movements than the restricted series described by Dr. Reid. Nevertheless, although that surgeon failed to grasp all the details of manipulation, as a method for the reduction of dislocations, yet modern surgery owes him a debt of gratitude for the invaluable contribution he made to science in directing attention to this subject, that can be but imperfectly paid by connecting his name with the cure of luxations by manipulation.

*Discussion.*—Dr. Conner remarked that he questioned the correctness of the statement of the essayist, that a dislocation of the thigh could take place at any point of the circle of the acetabulum without the consequence of a fracture. At the upper and outer border of the rim, for instance, the head of the femur would be brought against a firm, dense ligament, whose upper part consists of an aggregation of fibres, giving great strength to this locality. Morris claims that it was impossible for a dislocation to occur at this point. Certainly, although a few specimens are to be seen at different museums, simple dislocations without fracture are at this point very rare, especially if the limb be in a position of adduction. They could only occur when the limb is forced in a position of abduction; example, a bank of earth falling on an individual directly downwards, causing him to squat down; rupture of the capsular ligament takes place, the body thrown forwards with the knees inwards, and the head of the bone then forced behind the rim of the acetabulum. A disloca ion in the thyroid notch can frequently happen, by the head of the femur, instead of slipping into the cavity of the acetabulum, being thrown into the thyroid foramen. When the dislocation of the bone is upward and backward, the head of the femur in the process of reduction does not pass directly into its socket, but slides along the outer rim of the acetabulum and then slips in from below; if this rotation be carried too far the head of the femur may pass this point and enter the thyroid notch. This rotation of the head the speaker had frequently observed.

Dr. Bramble said that he could readily understand many facts viewed in the light of the principles adduced in the essayist's paper, which before were inexplicable. Those who had been dissatisfied with the movements described by Reid, while delighted with the method of reducing dislocations of the hip by manipulation, could see the reasons for such dissatisfaction. He would not go over the grounds already traversed by the essayist, but would content himself with a reference to a case recently reduced by him.

A child while coasting was violently thrown against a house. At the moment the violence was inflicted the thigh was flexed on the pelvis, the leg on the thigh. The dislocation was upward and backward. The left limb was the one injured, and on the outer side of the knee was a contusion, showing the point where the force was applied. Dr. Luff was the physician in attendance. When this gentleman became satisfied that a dislocation existed, the speaker's services were requested. The left lower extremity presented the characteristic evidence of a dislocation in the situation mentioned. Dr. Vance, at Dr. Bramble's request, accompanied him to this case, and assisted in its reduction. The doctor placed his hands on the hip of the patient, carefully isolated the great trochanter, neck and head of the femur, and diverted the attention of the patient while the speaker made the following manipulations: Standing on the right (uninjured) side of the patient, he grasped the leg by the ankle and below and behind the knee, flexed the leg on the thigh, rotated the femur a little inward, and then adducted, exerting firm traction upon and slightly flexing the femur. The head of the former moved in response to these manipulations, but did not enter the socket. Carefully maintaining all the ground they had gained by means of the hand grasping the ankle, he gave the leg a sharp restricted vibratory movement. In a moment the muscles of the thigh contracted, and the bone went home with an audible snap.

To those familiar with the restricted series of movements taught by Reid, it was not necessary to point out the difference between his (Reid's) method and the one the speaker adopted, which, as would be at once seen, was based upon the principles advocated in the paper just read.

Dr. Young thought that the positions in this discussion were too extreme. His belief was that in the great majority of cases the head of the bone in the upward and backward displacements escaped from the upward and backward portion of the capsular ligament. And, also, that its misplacement in the same position, by escaping through the lower and anterior margin, was possible, but that it often occurred was very improbable.

He could not concur in the position taken by one of the speakers, that the form of the acetabulum was such as to render the escape of the femur impossible without fracturing the edge of that cavity, unless the limb be carried to extreme adduction or a state of adduction that it would be impossible to obtain upon the living subject. For there were in this specimen (referring to the bone used by the essayist in demonstrating the mechanism in the reduction of dislocations at the hip joint)—and in all healthy ones this could be found—a regular and gradual inclination of the wall of the acetabulum from the centre to its outer margin. So that if the femur were slightly adducted when the person is being thrown with great force upon the feet or knee, it could be dislodged from the socket, without necessitating a fracture, to the upper margin of the acetabulum.

That it was possible to have dislocation upward and backward, the femur escaping through the capsule below and forward, was demonstrated by the lodgement of the bone upon the dorsum in the attempt at reduction from the pubes, or foramen thyroideum. And it was possible for either of these to have been originally dislocations upon the dorsum. The first case of reduction by manipulation that the speaker witnessed was by Dr. Swinburn, of Albany, in 1854—a dislocation upon the pubes. Having flexed the limb upon the pelvis, and whilst reflecting a moment as to the next movement which would most likely accomplish the reduction, the head escaped from its location on the pubes to the dorsum. In the attempt to remove it from this locality it passed into the foramen thyroideum, from which it was transplanted into the ischiatic notch, from which it was finally conveyed to the proper position. This was among the first, if not the first, cases reduced in that city by Reid's method. That the rupture of the capsule at the points referred to depend upon the position of the femur, whether in adduction or abduction was very evident. If we could be sure of the position in every case, it would go far to decide this question; but according to his experience it was very difficult to get a satisfactory history of these cases, as the accidents were usually so sudden and unexpected that the patient or those witnessing it could rarely or never give a satisfactory description of the occurrence. In attempting to demonstrate the cause or the probability of dislocations and fractures upon the dead subject, he could never obtain much satisfaction, for he found that anything that one might desire could be illustrated after the muscles and ligaments have been divided. The true causes of displacement, and resistance to reduction, could only be present in the living subject, with undivided tissues, except those that are actually involved in the lesion.

Dr. Young did not believe that the obstacle to reduction depended upon any one tissue or circumstance implicated. It was not the capsule grasping the neck of the femur where it escapes, or the ridge that surrounds the acetabulum, but it was their resistance, combined with the muscles, the influence of the latter being greater than all others combined. We have a displacement of most of the powerful muscles of the pelvis and femur, throwing them in vigorous contraction, binding the limb fixedly to the body, restricting motion to only a few directions, and presenting a feature so striking as to make it a diagnostic sign so frank that one would not be mistaken as to the character of the case one time out of ten.

In reduction by manipulation, the speaker always had a dread of failure; and every case had always been reduced at a moment when he was not expecting it. In one instance, when he was attempting to illustrate the facility with which reduction could be accomplished, instead of the head being deposited in the acetabulum it was thrown upon the pubes. This accident of throwing the dislocated head in a position not the one intended, rather than the acetabulum, was the result, as he thought, of a sudden and spasmodic action of the muscles when the head was directed toward the acetabulum and just mounting its margin; the operator, at this instant, holding the shaft too firmly, in a direction perpendicular to the cavity instead of letting it incline, so as to permit the head to reach its proper position.

